# Pest detection in dynamic environments: an adaptive continual test-time domain adaptation strategy

**DOI:** 10.1186/s13007-025-01371-y

**Published:** 2025-04-23

**Authors:** Rui Fu, Shiyu Wang, Mingqiu Dong, Hao Sun, Mohammed Abdulhakim Al-Absi, Kaijie Zhang, Qian Chen, Liqun Xiao, Xuewei Wang, Ye Li

**Affiliations:** 1 Shandong Facility Horticulture Bioengineering Research Center, Weifang University of Science and Technology, Weifang, 262700 China; 2 Sichuan International Studies University, Chongqing, 400031 China; 3 Beijing Institute of Technology, Beijing, 100081 China; 4 Department of Smart Computing, Kyungdong University, 46 4-gil, Bongpo, Giosung, 24764 Gangwon-do Korea; 5 Sichuan Technology and Business University, Chengdu, 610000 Sichuan China; 6 Southwest Jiaotong University, Chengdu, 610000 Sichuan China

**Keywords:** Pest detection, Unseen environment, Domain adaptation, Test-time adaptation, Self-supervised learning

## Abstract

Pest management is essential for agricultural production and food security, as pests can cause significant crop losses and economic impact. Early pest detection is key to timely intervention. While object detection models perform well on various datasets, they assume i.i.d. data, which is often not the case in diverse real-world environments, leading to decreased accuracy. To solve the problem, we propose the CrossDomain-PestDetect (CDPD) method, which is based on the YOLOv9 model and incorporates a test-time adaptation (TTA) framework. CDPD includes Dynamic Data Augmentation (DynamicDA), a Dynamic Adaptive Gate (DAG), and a Multi-Task Dynamic Adaptation Model (MT-DAM). Our DynamicDA enhances images for each batch by combining strong and weak augmentations. The MT-DAM integrates an object detection model with an image segmentation model, exchanging information through feature fusion at the feature extraction layer. During testing, test-time adaptation updates both models, continuing feature fusion during forward propagation. DAG adaptively controls the degree of feature fusion to improve detection capabilities. Self-supervised learning enables the model to adapt during testing to changing environments. Experiments show that without test-time adaptation, our method achieved a 7.6% increase in mAP50 over the baseline in the original environment and a 16.1% increase in the target environment. Finally, with test-time adaptation, the mAP50 score in the unseen target environment reaches 73.8%, which is a significant improvement over the baseline.

## Background

Insect pests are present throughout all developmental stages of cultivated plants, adversely impacting both the quality and quantity of agricultural produce. Pest infestations pose significant threats to the growth, development, yield, and quality of crops in agricultural production. Pests directly feed on plant leaves, stems, fruits, causing tissue damage and affecting the normal growth and development of plants. Some pests may carry pathogens during feeding, thereby spreading diseases to healthy plants and triggering more widespread disease outbreaks. Additionally, a novel insect species has severely affected salt marsh ecosystems, greatly accelerating the degradation of coastal salt marshes [1, 2]. Consequently, the direct damage caused by pests and the spread of diseases often severely affect plant yield and quality, reducing economic benefits for farmers[3, 4].

Traditionally, pest identification requires agricultural experts to conduct on-site observations. This approach may not detect problems promptly and reliance on experts necessitates a considerable level of professional knowledge. Consequently, some pests may not be identified in time or may be misdiagnosed. With technological advancements, new solutions have emerged that utilize machine learning and computer vision techniques to analyze images and identify pests on plants. This method not only improves the speed and accuracy of diagnosis but also reduces the need for professional agricultural expertise, making pest management more efficient and allowing for earlier interventions to protect crops from damage[5].

On the other hand, the advent of deep learning has changed the status quo of traditional pest detection and identification. Palazzetti et al. [6] propose a detection method for the brown-edged green moth using aerial remote sensing photos and neural networks, demonstrating the potential of deep learning in pest identification, integrating it with other technologies into pest management can achieve better results. Sorbelli et al. [7] use YOLO with RGB cameras and drones to detect the brown-edged green moth, achieving satisfactory results. Kusrini et al. [8] extend the pre-trained VGG-16 model to address dataset sparsity, reaching an accuracy rate of 76% on the test set. Amrani et al. [9] propose a multi-task model for pest detection, achieving accuracies of 75.77%, 66.39%, 70.01%, and 59% for detecting aphids in images of corn, rapeseed, rice, and wheat. Qing et al. [10] propose an insect imaging system with seven-fold cross-validation to improve recognition accuracy, achieving 97.5% average accuracy for four Lepidopteran rice pests. Kasinathan et al. [11] develop an insect detection algorithm using foreground extraction and contour recognition, with the CNN model achieving the highest accuracy rates (91.5% and 90.1%) on Deng [12] and Wu [13] datasets, respectively, and demonstrating lower computational times on practical agricultural datasets. To enhance model application in real scenarios and address multi-species and multi-growth-stage insect counting, Bereciartua-Pérez et al. [14] propose a deep learning-based multiclass and multi-growth-stage counting algorithm, achieving RMSE values from 0.89 to 4.47, MAE values from 0.40 to 2.15, and R$^{2}$ from 0.86 to 0.91 across various insect stages. Sangari et al. [15] introduce an ant colony optimization-based image segmentation method, which surpasses fuzzy C-means in quality for pest segmentation. Dewi et al. [16] apply ResNet-50 in a transfer learning paradigm, achieving 99.40% accuracy in pest detection. Suthakaran [17] proposes a CNN-based detection and classification system, achieving accuracies of 76.65%, 83.08%, 71.73%, 66.67%, and 65.17% for flea beetles, June beetles, ladybugs, squash beetles and chafers. Xie et al. [18] develop a swarm intelligence algorithm, SSAFS for plant disease detection, showing improved accuracy and fewer required features compared to other metaheuristics. Unlike traditional detection methods, the YOLO algorithm simultaneously predicts object positions and categories through a unified network, enabling fast detection. For instance, Gao et al. [19] develop a hybrid network for aphid counting, combining detection and density map estimation networks, and achieve low MAE and RMSE values across standard and high-density datasets. Kang et al. [20] use the YOLOv8 algorithm with simulated annealing and dropout regularization, enhancing pest detection accuracy and reducing overfitting. Experimental results indicate that the improved YOLOv8 algorithm surpasses YOLOv7 by 2% to 5% across sample sizes, peaking at 5.22% higher accuracy with 105 samples.

We note that existing pest identification methods typically focus on the analysis of static images. While this method is effective to some extent, it is based on a key assumption: the data is i.i.d.. This assumption limits the depth of research because it overlooks the dynamic changes in the agricultural environment. In reality, agricultural ecosystems are constantly changing,the development and variation of pests are influenced by a variety of environmental factors. Differences in environmental conditions across regions and changes over time in the same area can lead to significant shifts in data distribution. These distribution changes may significantly reduce the predictive accuracy of models when they encounter new environmental conditions or fluctuations. Therefore, to enhance the applicability and accuracy of models, future research needs to consider these dynamic factors and develop pest identification technologies that can adapt to environmental changes. For instance, under variable weather conditions such as cloudiness, rainfall, or extreme temperatures, the distribution and manifestation of pests may differ. These weather conditions might alter the appearance of pests, and such changes in characteristics may not be adequately represented in the model’s training data. Moreover, the emergence of new pest species or invasive alien species may introduce new patterns of features that existing models have not encountered before.

With the development of deep learning, researchers have made significant progress in addressing the challenge of distribution inconsistency between source and target domains through various data augmentation techniques. Recent research on cross-domain data augmentation shows a significant impact across various fields. For instance, Yu et al. [21] propose a novel cross-domain data augmentation method based on Domain-Adaptive Language Modeling (DALM), aimed at improving domain adaptation issues in Aspect-Based Sentiment Analysis (ABSA) tasks. Xu et al. [22] introduce an attention-guided cross-domain tumor image generation model (CDA-GAN), generating diverse samples through an information enhancement strategy to expand the dataset and improve performance in medical image diagnosis and treatment tasks. This work also introduces a novel strategy that uses synthetic images and difference images to enhance the feature learning capability of downstream segmentation models. Shen et al. [23] propose an online distillation method that enhances convolutional neural networks’ generalization through mixed sample augmentation methods, such as MixUp or CutMix. They make the first attempt to integrate CutMix into online distillation and propose a new online distillation-specific mixed sample enhancement strategy, named CutnMix.

**Fig. 1:**
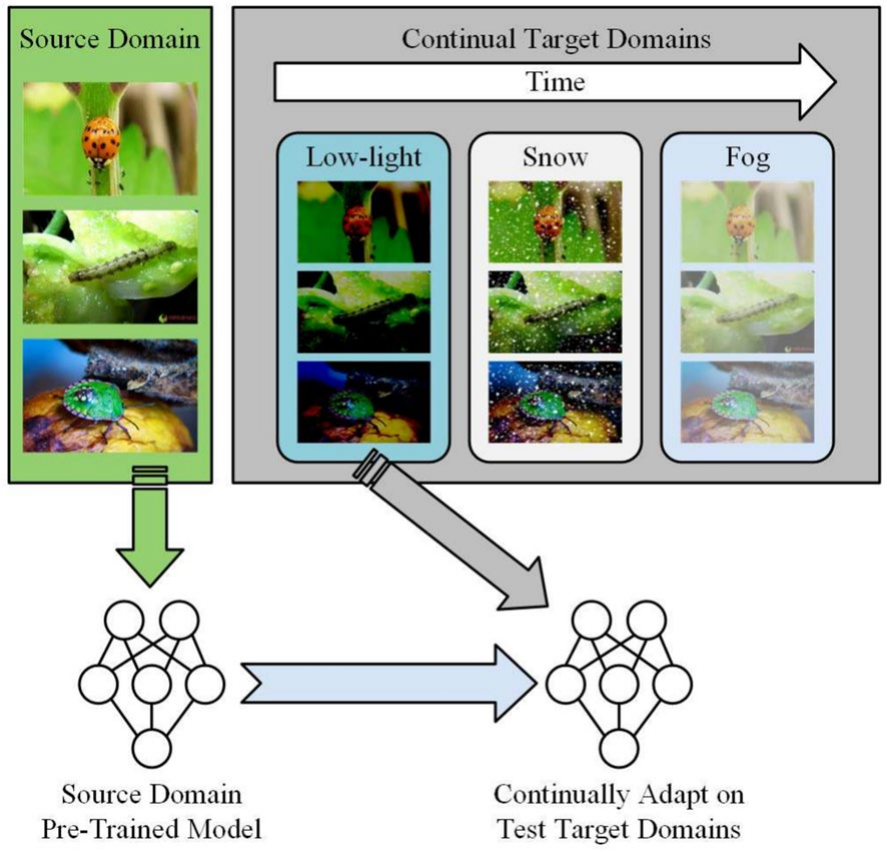
Diagram of Cross-Domain Continual Adaptation. The model undergoes initial pre-training on the source domain (natural images, green area) and sequentially adapts to multiple target domains (low-light, snow, fog, grey area), enhancing its generalization through continual adaptation

To prevent misdiagnoses and crop losses in dynamic environments, adaptive models like Test-Time Adaptation (TTA) provide accurate diagnoses and support timely interventions, essential for agricultural productivity and food security in changing environments. In the context of cross-domain testing, the adaptive process aims to enable models to quickly adapt and effectively handle the changes brought about by new environments after being trained in the initial environment, as illustrated in Fig. 1.

Researchers increasingly address the challenge of distributional inconsistency between source and target domains by employing TTA techniques, yielding promising results. TTA enables models to dynamically refine themselves with current target data, eliminating reliance on source data. Recent studies highlight the substantial impact of TTA across various fields. For instance, Sójka et al. [24] enhance an established self-training framework by incorporating a small memory buffer to improve model stability and dynamically adapt according to domain shift intensity. Lee et al.[25] design an ensemble algorithm with a self-training loss that differs from previous unsupervised domain adaptation methods, processing test data sequentially and online without source domain access at runtime. Shin et al. [26] develop an adaptive method known as Intermodal Pseudo-label Refinement (Inter-PR), which introduces consistent self-learning signals across multi-modal TTA scenarios for 3D semantic segmentation. Cohen et al. [27] emphasize the effectiveness of unsupervised and diversified TTA augmentation in anomaly detection, finding that it significantly improves detection accuracy and enhances model robustness by reducing false positives. Finally, Cui et al. [28] propose a knowledge distillation approach for TTA using a teacher-student network architecture, where the teacher is trained on augmented data from novel domains to achieve cross-domain generalization.

**Fig. 2:**
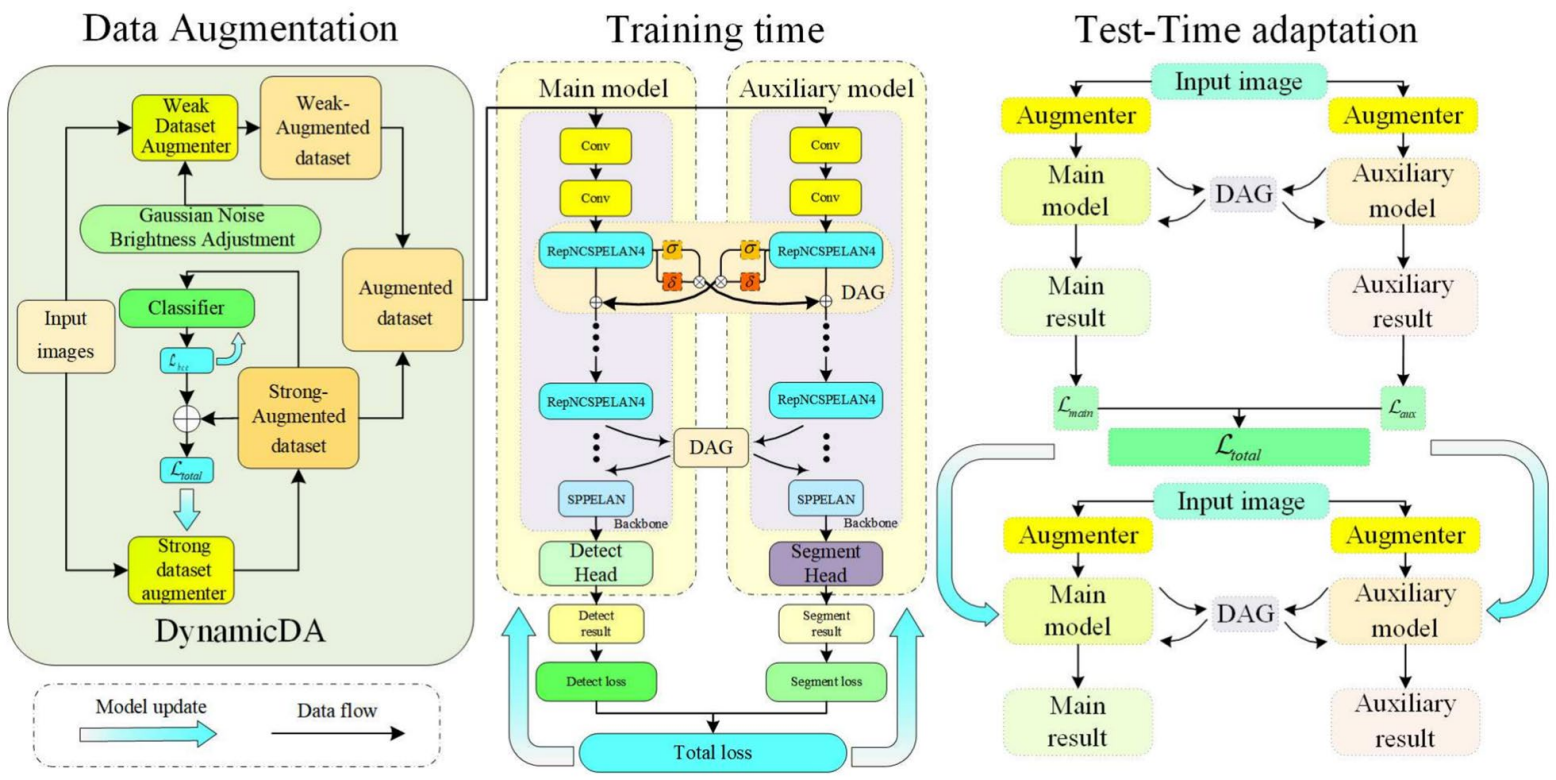
The proposed CDPD. The image ${{\textbf {X}}}$ is subjected to augmentation by a strong augmenter before being classified, yielding $\mathcal {L}_{bce}$. The total loss $\mathcal {L}_{total}$ accounts for batch and domain differences. $\mathcal {L}_{bce}$ is tasked with updating the classifier, while $\mathcal {L}_{total}$ refines the augmenter to maximize image divergence without inducing class changes. A weak augmenter operates in parallel as well. During training, the main and auxiliary models exchange knowledge through the DAG module that follows each RepNCSPELAN4 layer. Due to the presence of multiple RepNCSPELAN4 layers in the model, with a DAG module situated post each layer, we omit the repetitive sections in the illustration for clarity. The total loss updates main model, auxiliary model and DAG module. In testing, the pseudo-label with the highest confidence from both models is selected for further model refinement

In this work, inspired by the latest advances in cross-domain domain adaptation, we propose a method called CrossDomain-PestDetect (CDPD) based on the YOLOv9 model and the Test-Time Adaptation (TTA) framework. CDPD aims to address complex agricultural environment challenges and adapt to images with varying features and attributes in target environments. Specifically, our method comprises three key components: Dynamic Data Augmentation (DynamicDA), Dynamic Adaptive Gate (DAG), and Multi-Task Dynamic Adaptation Model (MT-DAM). During the data loading phase, we introduce an advanced dynamic augmentation technique consisting of two parts: a strong augmenter and a weak augmenter. In each training cycle, these augmenters generate a batch of images with diverse features as input data for the model. For model selection, we utilize YOLOv9 as both the main and auxiliary models. The main model adopts YOLOv9’s object detection architecture, while the auxiliary model is based on its image segmentation architecture. Although both share the same backbone, they differ in their network heads. We designate the output of the RepNCSPELAN4 layer in YOLOv9 as the key node for information exchange between the main and auxiliary models. This design enables feature fusion at different dimensions, allowing the models to learn from and absorb each other’s advantageous knowledge. During the training phase, the main and auxiliary models exchange beneficial knowledge through feature fusion. In the test-time adaptation phase, images undergo augmentation and are input into both models to obtain results from multiple augmented images. The result with the highest confidence is selected as the pseudo-label for calculating loss and updating the models. Similarly, feature fusion occurs between the main and auxiliary models during the test-time adaptation phase. However, feature fusion between the main and auxiliary models may lead to the transmission of incorrect information, potentially affecting model performance. To mitigate this issue, we incorporate a Dynamic Adaptive Gate, which allows the main and auxiliary models to fuse features through this module. The gate control module adaptively learns the degree of feature fusion during both training and testing, controlling the direction of feature fusion between the models and thereby enhancing detection capabilities. In summary, this work introduces the CDPD method, which aims to improve model detection accuracy and robustness in unseen environments, with the following contributions:

- We propose an innovative cross-domain pest detection method based on YOLOv9, aiming to improve the model’s detection accuracy under cross-domain conditions. This approach addresses a relatively underexplored area both domestically and internationally.
- CDPD utilizes a novel image augmentation algorithm that enhances the model’s adaptability to real environments by simulating variations in lighting, occlusion, and blur. This approach improves robustness through diversified training samples, maintaining sensitivity to source data while adapting to target domain characteristics, thereby enhancing generalization ability.
- CDPD incorporates a Dynamic Adaptive Gate (DAG) algorithm during feature fusion, which dynamically adjusts fusion trade-off parameters based on model performance across different environments and tasks. This algorithm intelligently modulates the degree of feature fusion by learning task correlations, thereby optimizing model performance under diverse conditions. Experimental results demonstrate the effectiveness of CDPD in practical applications, achieving an mAP50 of 73.8% in the target environment after test-time adaptation.
- CDPD employs a MultiTask Dynamic Adaptation Model (MT-DAM), comprising a main model for object detection and an auxiliary model for image segmentation. These two branches exchange information through DAG feature fusion, mutually learning beneficial knowledge to enhance model accuracy. During the testing phase, MT-DAM utilizes test-time adaptation by augmenting images to generate 16 variants, which are then input into both the main and auxiliary models to obtain multiple results. The result with the highest confidence is selected as the pseudo-label, and loss is calculated using these results and pseudo-labels to update the models (Fig. 2).

## Method

**Fig. 3:**
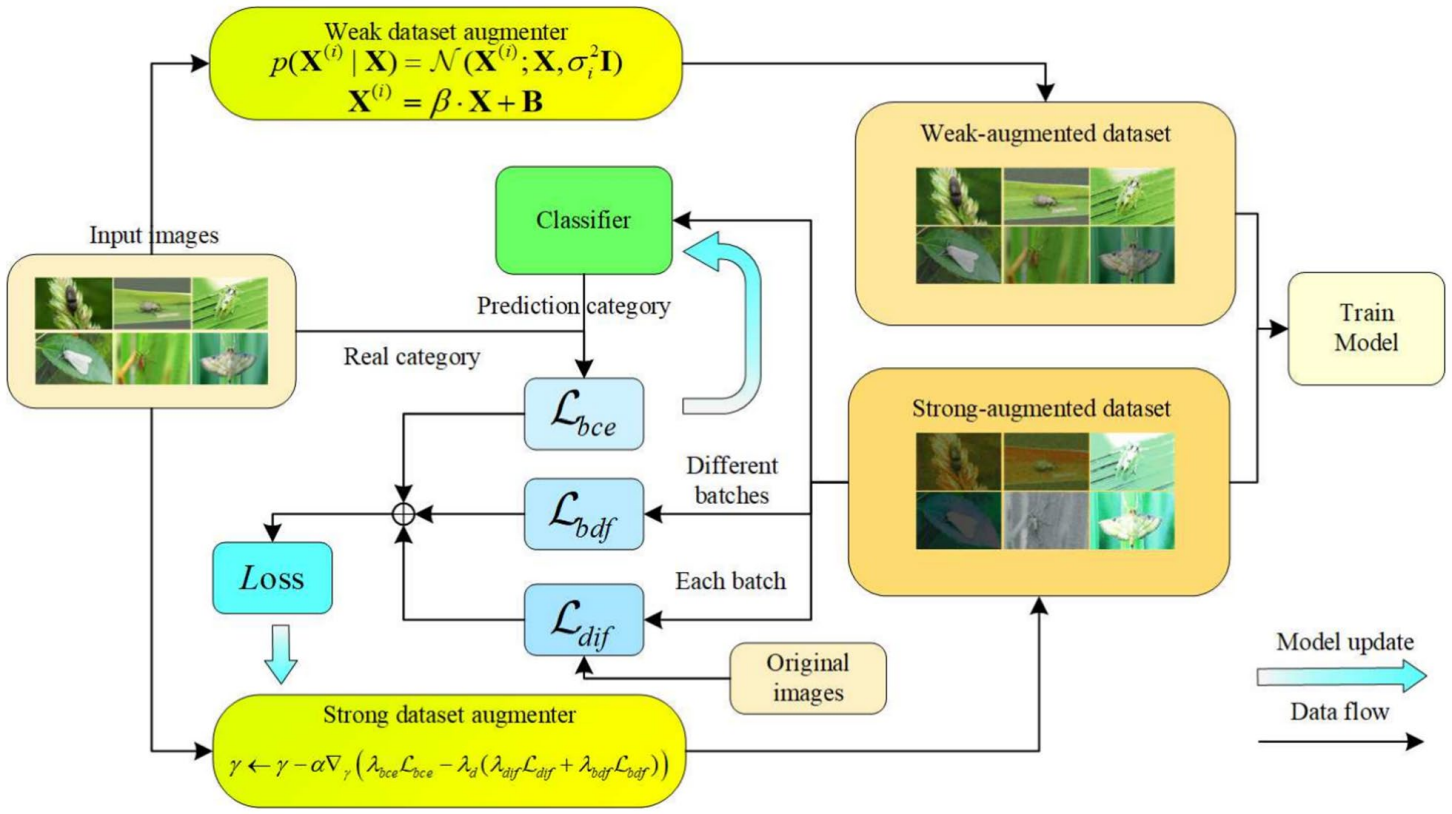
The proposed dynamic data augmentation. The image ${{\textbf {X}}}$ undergoes augmentation by a strong augmenter and is classified by a classifier to compute the binary cross-entropy loss $\mathcal {L}_{bce}$, which updates the classifier. Batch difference loss $\mathcal {L}_{bdf}$ and domain difference loss $\mathcal {L}_{dif}$ are calculated and combined to update the strong augmenter. After training, the last 3 rounds of images form the strong-augmented dataset. The input image is also augmented by a weak augmenter for 3 rounds, tripling the dataset size to create the weak-augmented dataset. Both augmented datasets and the original images are used as inputs for the model

To ensure that the model maintains accuracy comparable to the source domain across diverse environments without significant decline, we introduce an advanced dynamic data augmentation technique during the model’s data loading phase. This technique comprises two components: a strong augmenter and a weak augmenter. The strong augmenter generates images that are sent to the classifier for classification and optimized to minimize category loss, ensuring that the newly generated data provide practical value for model training. Additionally, we calculate a difference loss based on the strong augmenter’s output, which includes the differences between images within each batch and between each batch and the original images. By maximizing these two losses to update the model, the strong augmenter is encouraged to produce images that differ more from the original environment and possess more diverse features. Concurrently, the weak augmenter applies minor augmentations to complement images that are less altered from the original environment. DAG module controls the intensity of information exchange between the main and auxiliary models by leveraging the model’s RepNCSPELAN4 layer and calculating weights through a fully connected layer, representing the strength of information transfer between branches. In each DAG module following the RepNCSPELAN4 layer, weight calculations occur, and these weights, being learnable parameters, are continuously updated via gradient descent during training. These weights regulate the degree of feature fusion between branches through element-wise multiplication, fine-tuning the information flow and ensuring that the model maintains excellent performance across various environments. During each training epoch, the augmenters generate a batch of images with diverse features as input data. The main and auxiliary models operate in parallel, exchanging information through the DAG module, which allows them to learn beneficial knowledge from each other and enhance detection performance in complex environments. In the testing phase, images undergo augmentation and are input into both models. The models fuse features and exchange information via the DAG module during forward propagation. After processing multiple augmented images, the result with the highest confidence is selected as a pseudo-label, and the loss is calculated using these results and pseudo-labels to update both models (Fig.3).

### Dynamic data augmentation

With the development of deep learning, existing models have achieved good results in detection capabilities within the source domain of the training set. However, in the field of pest detection, the model’s detection capabilities in new domains may decline significantly due to changes in environmental and lighting conditions. Existing studies have demonstrated that image enhancement plays a significant role in generalization[29, 30]. To enhance the model’s cross-domain detection capabilities, we apply learnable dynamic augmentation to the original training set at the beginning of each batch and save the results from the final 3 rounds, producing 3 times the augmented images. At the same time, the weak augmenter generates a weak-augmented dataset by injecting Gaussian noise and adjusting brightness. These two newly generated datasets, along with the original dataset, are used as inputs to the model.

#### Strong augmenter

To generate cross-domain images with different characteristics from the original images, we introduce a generator, the ResNetGenerator. The ResNetGenerator is a generator network constructed based on ResNet blocks, consisting of multiple downsampling/upsampling operations and ResNet blocks, typically used for image-to-image translation tasks such as style transfer. This makes it suitable as our generator for producing cross-domain images.

To regulate the degree of divergence between the output images from the strong augmenter and the original images, ensuring that the generated images remain within the original categories, we have integrated a learnable classifier into the strong augmenter. This classifier utilizes a ResNet101 network, which has been pre-trained and subsequently fine-tuned on a pest dataset. It processes the data augmented by the strong augmenter and yields classification results. It calculates the difference between the augmented data and the original data using binary cross-entropy loss. Both the strong augmenter and the classifier are updated by minimizing the binary cross-entropy loss $\mathcal {L}_{bce}$:

1 $$\begin{aligned} \mathcal {L}_{bce} = -\frac{1}{N K} \sum _{k=1}^{K} \left[ y_{k} \cdot \log (\hat{p}_{k}) + (1 - y_{k}) \cdot \log (1 - \hat{p}_{k}) \right] \end{aligned}$$

where *N* represents the number of categories in the dataset, and *K* is the total number of batches generated by the dynamic augmentation. Here, $\hat{p}_{k}$ is the predicted probability of the classifier for the *k*-th strong augmenter, and $y_{k}$ is the correct binary label. In cases where an image contains multiple categories, the labels $y_{k}$ can be extended to a multi-label format, where each label indicates the presence or absence of a category. During the training process, both the strong augmenter and the classifier are updated to minimize the binary cross-entropy loss $\mathcal {L}_{\text {bce}}$, ensuring the consistency of the augmented data with the original data categories and preventing them from deviating to other categories.

On the premise of ensuring that the category of the augmented data remains unchanged, in order to enable the model to learn knowledge of more complex environments, we propose to maximize the original augmented image difference loss $\mathcal {L}_{dif}$ to generate augmented data for more complex environments:

2 $$\begin{aligned} \mathcal {L}_{dif} = \sum _{i=1}^{n} \left( \frac{1}{M \times N \times C} \sum _{m=1}^{M} \sum _{n=1}^{N} \sum _{c=1}^{C} ({\textbf {X}}^{ori}_{i,m,n,c}- {\textbf {X}}^{aug}_{i,m,n,c})^2 \right) , \end{aligned}$$

where *n* is the total number of original and augmented image pairs, *M*, *N*, *C* represent the height, width, and channel number of the image, respectively, ${\textbf {X}}^{ori}_{i,m,n,c}$ and ${\textbf {X}}^{aug}_{i,m,n,c}$ are the pixel values of the *i*-th original image and augmented image at position (*m*, *n*) and channel *c*.

Furthermore, although maximizing the original augmented image difference loss $\mathcal {L}_{dif}$ can ensure that the original and augmented images are not in the same environment, to prevent the augmented images from gradually fixing in a specific environment other than the original environment, we also propose to maximize the difference loss of augmented images per batch $\mathcal {L}_{bdf}$ to maximize the difference of the augmented data generated per batch:

3 $$\begin{aligned} \mathcal {L}_{bdf} = \frac{2}{n(n - 1)} \sum _{i=0}^{n-1} \sum _{\begin{array}{c} j=i+1 \\ j \ne i \end{array}}^{n} \left( \frac{1}{M \times N \times C} \sum _{m=1}^{M} \sum _{n=1}^{N} \sum _{c=1}^{C} ({\textbf {X}}^{ori}_{i,m,n,c} - {\textbf {X}}^{aug}_{i,m,n,c})^2 \right) , \end{aligned}$$

where *n* represents the total number of images in the augmented image set, *M*, *N*, *C* are the height, width, and channel number of the image. Maximizing the difference loss of augmented images per batch $\mathcal {L}_{bdf}$ ensures that the augmented images are not limited to a specific environment, augmenting the diversity of the augmented images.

For dynamic augmentation, the parameters $\gamma$ are updated by minimizing the classification loss $\mathcal {L}_{bce}$, maximizing the original augmented image difference loss $\mathcal {L}_{dif}$, and maximizing the difference loss of augmented images per batch $\mathcal {L}_{bdf}$, ensuring the generation of as diverse as possible augmented images, and augmented images from different environments for the model:

4 $$\begin{aligned} \gamma \leftarrow \gamma -\alpha \nabla _{\gamma }\left( \lambda _{bce} \mathcal {L}_{bce}-\lambda _{d}(\lambda _{dif} \mathcal {L}_{dif}+\lambda _{bdf} \mathcal {L}_{bdf})\right) , \end{aligned}$$

where $\alpha = 0.01$ is the learning rate of the strong augmenter, $\lambda _{bce} = 0.4$, $\lambda _{d} = 0.6$, $\lambda _{dif} = 0.5$, $\lambda _{bdf} = 0.5$ are the weight parameters of the respective losses, used to adjust the level of diversity of the augmented data.

#### Weak augmenter

*Gaussian Noise* In deep learning, models must handle various defects and noise present in real-world data[31]. Gaussian noise is a common type of noise that plays a crucial role in the training process by enhancing the model’s generalization ability and performance across different datasets[32]. By introducing Gaussian noise during training, defects are simulated, prompting the model to focus on key data features rather than relying on accidental correlations. Specifically, Gaussian noise is added by imposing random disturbances on the pixel values of the image, following a Gaussian distribution. For a given image ${{\textbf {X}}}$, its augmented version ${{\textbf {X}}}^{(i)}$ is generated using the following formula:

5 $$\begin{aligned} p({{\textbf {X}}}^{(i)}|{{\textbf {X}}}) = \mathcal {N}({{\textbf {X}}}^{(i)}; {{\textbf {X}}}, \sigma _i^2 {{\textbf {I}}}) , \end{aligned}$$

where $\mathcal {N}({{\textbf {X}}}^{(i)}; {{\textbf {X}}}, \sigma _i^2 {{\textbf {I}}})$ represents a Gaussian distribution centered on the original image ${{\textbf {X}}}$, with the covariance matrix composed of $\sigma _i^2$ multiplied by the identity matrix ${{\textbf {I}}}$. This indicates that the augmented image ${{\textbf {X}}}^{(i)}$ is affected by Gaussian noise with a standard deviation of $\sigma _i$. In our experiments, four different standard deviations $\sigma = [4, 8, 12, 16]$ are selected to investigate the impact of varying noise levels on model performance.

*Brightness Adjustment* Variations in brightness due to different lighting conditions can significantly affect object recognition in images. To address this issue, we employ brightness adjustment techniques to simulate real-world lighting changes and enhance the model’s adaptability. This method adjusts the brightness of the image by multiplying the original image ${{\textbf {X}}}$ by a scaling factor $\beta$ and adding an offset ${{\textbf {B}}}$:

6 $$\begin{aligned} {{\textbf {X}}}^{(i)} = \beta \cdot {{\textbf {X}}} + {{\textbf {B}}}, \end{aligned}$$

where the scaling factor $\beta$ is uniformly distributed between 0.7 and 1.3, regulating image brightness, and the offset term ${{\textbf {B}}}$ ranges from $-0.3$ to 0.3, fine-tuning the color balance. These adjustments enhance the model’s performance under varying lighting conditions by ensuring robustness to changes in brightness.

### Dynamic adaptive gate module

**Fig. 4:**
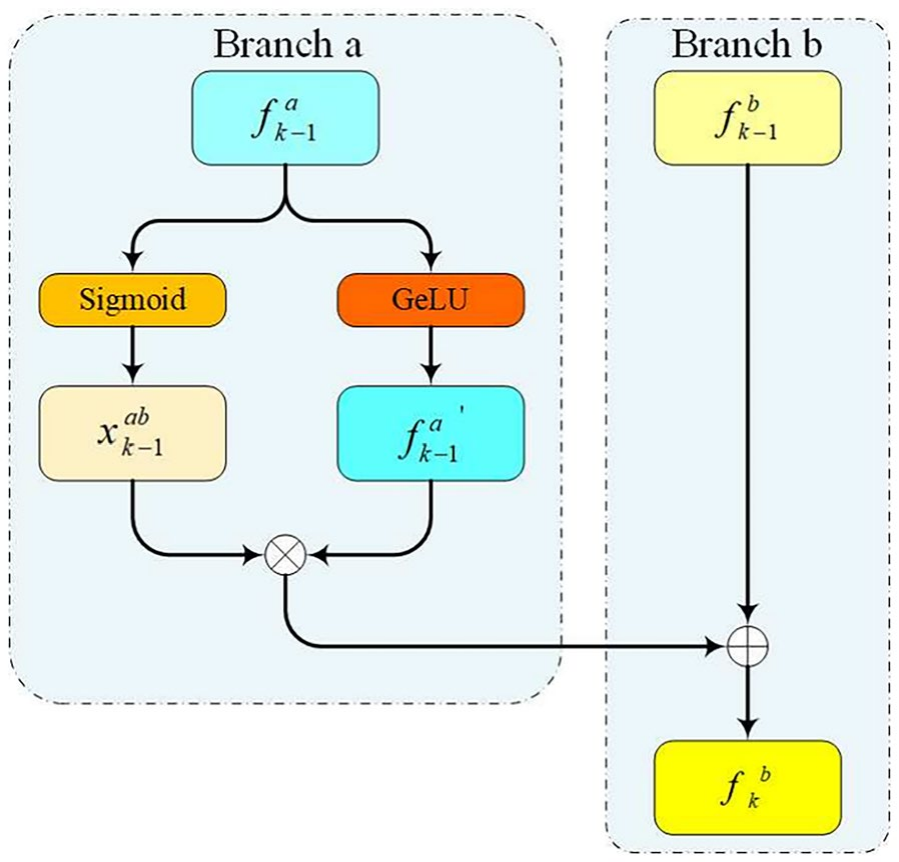
Dynamic Adaptive Gate Module. In the diagram, $\otimes$ denotes element-wise multiplication, and $\oplus$ denotes addition

The Dynamic Adaptive Gate Module is a pivotal component in our design, managing the flow of information between different tasks in a multi-task learning environment[33]. By integrating the Dynamic Adaptive Gate Module, we effectively control feature transfer between task branches, ensuring that only useful and accurate information is shared while preventing the exchange of invalid or erroneous data that could degrade performance[34]. In our implementation, each Dynamic Adaptive Gate Module calculates message passing weights between task branches through a fully connected layer. These weights adjust and fuse features from different branches, generating richer and more targeted feature representations: (FIg. 4).

7 $$\begin{aligned} x_{k-1}^{ab} = \sigma (W_{k-1}^a f_{k-1}^{a} + b_{k-1}^{a}), \end{aligned}$$

where $x_{k-1}^{ab}$ represents the message passing strength from branch $a$ to branch $b$, $f_{k-1}^{a}$ is the output feature of branch $a$ at layer $k-1$, $W_{k-1}^a$ and $b_{k-1}^{a}$ are the learnable weights and biases, respectively, and $\sigma$ is the sigmoid activation function. Using these weights and the previous layer’s features, we calculate the output features of branch $b$ at the current layer:

8 $$\begin{aligned} f_k^b = \sigma (W_{k-1}^a f_{k-1}^{a} + b_{k-1}^{a}) \otimes o_{k-1}^a \oplus f_{k-1}^b, \end{aligned}$$

Here, $f_k^b$ represents the output features of branch $b$ after fusing features from branch $a$, and $\odot$ denotes element-wise multiplication. The sigmoid function normalizes the weights between 0 and 1, controlling the degree of feature fusion between branches. This mechanism allows dynamic adjustment of the feature fusion process based on learned weights, enhancing the model’s adaptability to different tasks and environments:

9 $$\begin{aligned} o_{k-1}^a&= \delta (U_{k-1}^{a} f_{k-1}^{a} + e_{k-1}^{a}), \end{aligned}$$

10 $$\begin{aligned} f_k^b&= x_{k-1}^{ab} \otimes o_{k-1}^a \oplus f_{k-1}^b, \end{aligned}$$

where $f_{k-1}^b$ is the output feature of branch $b$ at layer $k-1$, $\otimes$ denotes element-wise multiplication, $\delta$ represents the GeLU (Gaussian Error Linear Unit) activation function, and $U_{k-1}^{a}$ and $e_{k-1}^{a}$ are learnable weights and biases, respectively. $f_k^b$ serves as input to the next layer. During training, the weights and biases of the fully connected layer are automatically updated based on the gradient of the loss function. This adaptive learning capability allows the model to continuously optimize its parameters to adapt to changing data distributions and task requirements.

### MultiTask dynamic adaptation model (MT-DAM)

To enhance the model’s precision and generalization capability, we propose an innovative multi-task learning model based on YOLOv9, named the MultiTask Dynamic Adaptation Model (MT-DAM). This model comprises a main model responsible for object detection and an auxiliary model tasked with image segmentation. This design leverages the complementarity between object detection and image segmentation tasks, improving the model’s adaptability to different visual tasks by sharing low-level feature representations. The main model identifies objects and predicts their locations, while the auxiliary model refines this process by enhancing the detection model’s ability to recognize details through pixel-level information learned from segmentation tasks.

#### Domain generalization learning

In the training phase, the core structure of the MT-DAM model consists of two parts: the main model adopts YOLOv9’s object detection architecture, while the auxiliary model is based on YOLOv9’s image segmentation architecture. Although both share the same backbone network, they differ in their network heads. We select the output of the RepNCSPELAN4 layer in YOLOv9 as the key node for information exchange between the main and auxiliary models. This design allows feature fusion at different feature dimensions, enabling the models to learn from and absorb each other’s strengths.

Information exchange is facilitated through the DAG module, as described in section Dynamic adaptive gate module. The DAG module contains learnable parameters that regulate the intensity of information exchange, promoting the transfer of beneficial information while suppressing the propagation of erroneous data. Specifically, during training, the main and auxiliary models input the same image but use different labels. After passing through each RepNCSPELAN4 layer, the main and auxiliary models fuse their feature maps via the DAG module and use the fused features as inputs for subsequent processing. This process is represented by the following formulas:

11 $$\begin{aligned} f_k^{main}= & \sigma (W_{k-1}^{aux} f_{k-1}^{aux} + b_{k-1}^{aux}) \odot \delta (U_{k-1}^{aux} f_{k-1}^{aux} + e_{k-1}^{aux}) + f_{k-1}^{main}, \end{aligned}$$

12 $$\begin{aligned} f_k^{aux}= & \sigma (W_{k-1}^{main} f_{k-1}^{main} + b_{k-1}^{main}) \odot \delta (U_{k-1}^{main} f_{k-1}^{main} + e_{k-1}^{main}) + f_{k-1}^{aux}. \end{aligned}$$

Here, $f_k^{main}$ and $f_k^{aux}$ represent the features of the main and auxiliary models after feature fusion, serving as inputs for the next step of each model. $W$ and $U$ denote trainable weights, while $b$ and $e$ denote trainable biases. $f_{k-1}^{main}$ and $f_{k-1}^{aux}$ are the output features of the main and auxiliary models at the RepNCSPELAN4.

The DAG module learns feature interaction patterns between different task branches, automatically optimizing the weight and bias parameters during model updates. This mechanism provides a channel for information exchange between the object detection and image segmentation tasks, enabling the object detection model to more precisely recognize object locations and the image segmentation model to more accurately predict object boundaries. Consequently, the MT-DAM model demonstrates excellent performance across different tasks.

During the training process, two independent main trunk networks are responsible for extracting features from images and use different head networks for the training of object detection and image segmentation tasks. As the training progresses, the model considers the optimization objectives for both tasks. For the main model, category loss, localization loss and confidence loss are used:

13 $$\begin{aligned} \begin{aligned} \mathcal {L}_{main}^{train}&= -\frac{1}{N_{cls}} \sum _{i=1}^{N_{cls}} (t_{cls,i} \cdot \log (p_{cls,i}) + (1 - t_{cls,i}) \cdot \log (1 - p_{cls,i})) \\&\quad + 1 - IoU(pred\_boxes, true\_boxes) \\&\quad -\frac{1}{N_{conf}} \sum _{i=1}^{N_{conf}} (t_{conf,i} \cdot \log (p_{conf,i}) + (1 - t_{conf,i}) \cdot \log (1 - p_{conf,i})) \end{aligned} \end{aligned}$$

where $\mathcal {L}_{main}^{train}$ represents the total loss function of the main task model, $N_{cls}$ denotes the number of positive samples in the category loss, $t_{cls,i}$ represents the target category label of the *i*-th sample (0 or 1), $p_{cls,i}$ represents the model’s predicted probability of that category, *IoU* represents the Intersection over Union, used to measure the overlap between predicted and true bounding boxes, $pred\_boxes$ denotes the predicted bounding boxes, $true\_boxes$ denotes the true bounding boxes, $N_{conf}$ represents the total number of samples in the confidence loss, $t_{conf,i}$ represents the target existence label of the *i*-th sample (0 or 1) and $p_{conf,i}$ represents the model’s predicted confidence of target existence. For the auxiliary model, the segmentation loss is used:

14 $$\begin{aligned} \mathcal {L}_{aux}^{train}=1 - \frac{2 \sum _{i=1}^{H} \sum _{j=1}^{W} p_{ij} t_{ij}}{\sum _{i=1}^{H} \sum _{j=1}^{W} p_{ij}^2 + \sum _{i=1}^{H} \sum _{j=1}^{W} t_{ij}^2} \end{aligned}$$

where $\mathcal {L}_{aux}^{train}$ represents the total loss of the auxiliary model, *H* and *W* are the height and width of the image, respectively, $t_{ij}$ is the label indicating whether the pixel at position (*i*, *j*) belongs to the target (0 or 1), and $p_{ij}$ is the model’s predicted probability that the pixel at position (*i*, *j*) belongs to the target. Adding the total losses of the two models gives the final loss $\mathcal {L}_{total}^{train}$:

15 $$\begin{aligned} \mathcal {L}_{total}^{train} = \mathcal {L}_{main}^{train} + \lambda ^{train} \mathcal {L}_{aux}^{train} \end{aligned}$$

where the weight coefficient $\lambda ^{train}$ is responsible for adjusting the weight of the auxiliary model loss in the total loss. Since the two tasks are feature-fused through a dynamic adaptive gating module during the forward propagation process, the parameters of the main task, auxiliary task, and gating module are included in the same optimizer, and the final loss is used for backpropagation and parameter update to achieve dynamic control of feature fusion:

16 $$\begin{aligned} \theta _{main}= & \theta _{main} - \epsilon \nabla _{\theta _{main}} \mathcal {L}_{total}^{train} \end{aligned}$$

17 $$\begin{aligned} \theta _{aux}= & \theta _{aux} - \epsilon \nabla _{\theta _{aux}} \mathcal {L}_{total}^{train} \end{aligned}$$

18 $$\begin{aligned} \theta _{DAG}= & \theta _{DAG} - \epsilon \nabla _{\theta _{DAG}} \mathcal {L}_{total}^{train} \end{aligned}$$

where $\theta _{main}$ represents the parameters of the main task model, $\theta _{aux}$ represents the parameters of the auxiliary task model, $\theta _{DAG}$ represents the parameters of the dynamic adaptive gating module, $\epsilon$ represents the learning rate, and $\nabla _{\theta _{main}}$, $\nabla _{\theta _{aux}}$, $\nabla _{\theta _{DAG}}$ represent the gradients of the main task model parameters, auxiliary task model parameters and DAG module parameters. The specific training process is shown in Algorithm 1.

**Algorithm 1:**
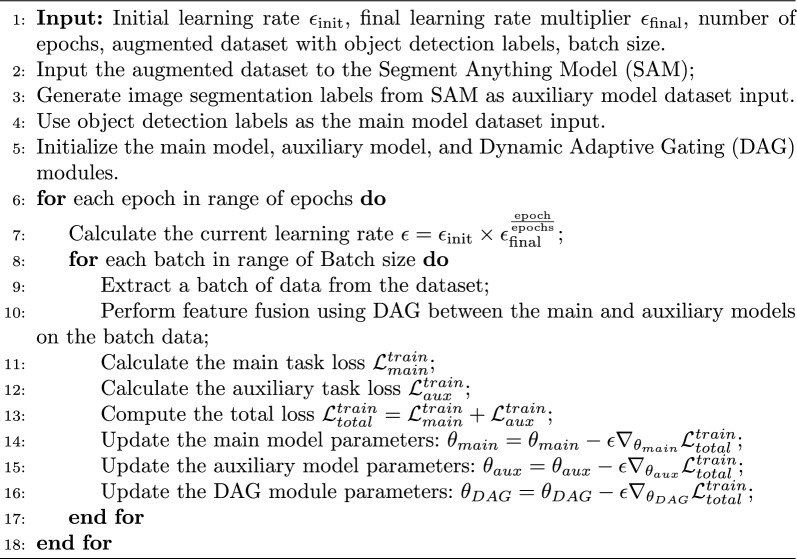
Dual-Task Learning for Object Detection and Image Segmentation

Notably, the auxiliary model’s image segmentation task annotations are automatically generated by the Segment Anything Model (SAM)[35, 36], significantly improving annotation efficiency and ensuring annotation consistency and accuracy. As an advanced image segmentation tool, SAM quickly generates high-quality segmentation masks based on existing object detection labels.

Specifically, SAM utilizes pre-trained deep learning techniques to identify and segment various object types. This pre-training grants the model strong generalization capabilities, allowing it to process new images and objects without targeted annotation data. During annotation, SAM automatically generates corresponding segmentation masks based on bounding boxes or other cues from the object detection model. This process accelerates annotation speed and reduces errors and inconsistencies that may occur during manual annotation, ensuring high-quality and consistent data annotation. Therefore, we only need to prepare images and their object detection labels to directly input data into the model for multi-task training.

#### Test-time Adaptation

The Continuous Test-Time Adaptation (CTTA) capability of the MultiTask Dynamic Adaptation Model (MT-DAM) serves as a core feature. This capability enables the model to dynamically adapt to changes in input images during the testing phase, thereby enhancing the model’s accuracy and generalization ability. Specifically, upon receiving a new input image, the MT-DAM model performs a series of data augmentation operations, generating 16 processed images that are then fed into the main and auxiliary models. During the testing phase data augmentation, two methods are employed: Gaussian noise and brightness adjustments, as illustrated by the following formulas:

19 $$\begin{aligned} I'(x, y)&= I(x, y) + \mathcal {N}(0, \alpha ^2), \end{aligned}$$

20 $$\begin{aligned} I'(x, y)&= L \cdot I(x, y), \end{aligned}$$

where $I(x, y)$ denotes the pixel value of the original image at position $(x, y)$, and $I'(x, y)$ represents the pixel value after processing. $\mathcal {N}(0, \alpha ^2)$ is a Gaussian distribution random variable with mean 0 and variance $\alpha ^2$, representing the Gaussian noise added to the image. $\alpha$ signifies the standard deviation of the Gaussian noise, controlling the noise intensity. $L$ denotes the brightness change factor, used to adjust the image brightness. These two enhancement methods can be applied simultaneously, as shown below:

21 $$\begin{aligned} I''(x, y) = (I(x, y) + B) + \mathcal {N}(0, \alpha ^2), \end{aligned}$$

where $I''(x, y)$ denotes the pixel value of the image after both enhancement methods have been applied at position $(x, y)$. This enhancement approach not only provides sufficient augmentation to the images but also avoids excessive complexity, preventing a significant increase in the number of parameters during the testing phase.

During the testing phase, the MT-DAM model utilizes the DAG module for feature fusion to ensure continuous interaction and learning between the main and auxiliary models during inference. Similar to the training phase, the DAG module dynamically adjusts the information flow between the main and auxiliary models through its learnable parameters, effectively fusing their respective features. When presented with new input data, the DAG module modulates the intensity of information exchange based on the current image’s features, ensuring that useful features are fully shared while preventing the propagation of noise or irrelevant information. Specifically, during the CTTA process, the main and auxiliary models process their respective tasks independently while achieving feature interaction and enhancement through the DAG module. This mechanism allows the object detection model to utilize detailed information from the image segmentation task to improve precise object localization, while the image segmentation model benefits from global location information provided by object detection, further optimizing segmentation results. Therefore, the DAG feature fusion mechanism during the testing phase ensures that the model maintains strong adaptability and high-precision performance across different scenarios and data distributions.

In the final stage of the testing process, the main and auxiliary models select the result with the highest confidence as the pseudo-label and calculate the difference between their outputs and the pseudo-label. This step measures the consistency of each model’s output with the target pseudo-label, thereby guiding model updates. Specifically, the main and auxiliary models calculate loss functions based on their own prediction results and the pseudo-labels. These loss functions reflect the deviation of each model’s output from the ideal target. Subsequently, the main and auxiliary models update their parameters based on these loss values to minimize the loss and enhance model performance. Specifically, the main model’s loss $\mathcal {L}_{main}$ is calculated by the following formulas:

22 $$\begin{aligned} & \mathcal {L}_{cls}^{main} = -\sum _{i=1}^{N} \sum _{c=1}^{C} y_{ic} \log (p_{ic}), \end{aligned}$$

23 $$\begin{aligned} & \mathcal {L}_{loc}^{main} = \sum _{i=1}^{N} \sum _{j=1}^{4} \left( t_{ij} - v_{ij} \right) ^2, \end{aligned}$$

24 $$\begin{aligned} & \mathcal {L}_{conf}^{main} = -\sum _{i=1}^{N} \left( y_i \log (p_i) + (1 - y_i) \log (1 - p_i) \right) , \end{aligned}$$

25 $$\begin{aligned} & \mathcal {L}_{main} = \mathcal {L}_{cls}^{main} + \mathcal {L}_{loc}^{main} + \mathcal {L}_{conf}^{main}, \end{aligned}$$

where $\mathcal {L}_{cls}^{main}$ represents the classification loss of the main model, measuring the difference between the model’s predicted category probabilities and the true labels. $N$ denotes the total number of samples, $C$ represents the total number of categories, $y_{ic}$ is the category pseudo-label indicating whether the $i$-th sample belongs to category $c$, and $p_{ic}$ is the probability that the model predicts the $i$-th sample belongs to category $c$. $\mathcal {L}_{loc}^{main}$ represents the localization loss of the main model, measuring the difference between the model’s predicted bounding box positions and the true bounding box positions. $t_{ij}$ is the bounding box position parameter predicted by the model for the $i$-th sample, and $v_{ij}$ is the pseudo-label bounding box position parameter. $\mathcal {L}_{conf}^{main}$ represents the confidence loss of the main model, measuring the difference between the model’s confidence in the presence of an object and the true situation. $y_i$ is the pseudo-label indicating whether the $i$-th sample contains an object, and $p_i$ is the probability that the model predicts the $i$-th sample contains an object. Finally, $\mathcal {L}_{main}$ is the total loss of the main model, the sum of classification loss, localization loss, and confidence loss, reflecting the model’s overall performance.

The auxiliary model’s loss $\mathcal {L}_{aux}$ is calculated by the following formula:

26 $$\begin{aligned} \mathcal {L}_{aux} = \sum _{i=1}^{N} \sum _{j=1}^{H \times W} \left( s_{ij} - g_{ij} \right) ^2, \end{aligned}$$

where $\mathcal {L}_{aux}$ represents the total loss of the auxiliary model, focusing on evaluating the model’s performance on the image segmentation task. This loss function measures segmentation accuracy by calculating the difference between the model’s predicted segmentation mask and the true segmentation mask. In this formula, $N$ represents the total number of samples, $H \times W$ represents the total number of pixels in the image (i.e., the product of image height $H$ and width $W$). $s_{ij}$ is the segmentation mask value predicted by the model for the $i$-th sample at pixel position $j$, and $g_{ij}$ is the pseudo-label segmentation mask value at the corresponding position. By minimizing $\mathcal {L}_{aux}$, the auxiliary model learns more precise segmentation boundaries, thereby achieving better performance on the image segmentation task. This loss calculation method encourages the model to learn at the pixel level, ensuring high-quality segmentation results.

Finally, the total loss is the weighted sum of the losses of the main model and the auxiliary model, which comprehensively considers the performance of both models to ensure their collaborative optimization. This can be expressed as:

27 $$\begin{aligned} \mathcal {L}_{total} = \mathcal {L}_{main} + \lambda \mathcal {L}_{aux}, \end{aligned}$$

where $\mathcal {L}_{main}$ and $\mathcal {L}_{aux}$ are calculated as the differences between the predicted outputs of the main model and the auxiliary model and the pseudo-labels, respectively. The weight coefficient $\lambda$ adjusts the impact of the auxiliary model’s loss in the total loss to ensure a balanced contribution from both models during training.

**Algorithm 1:**
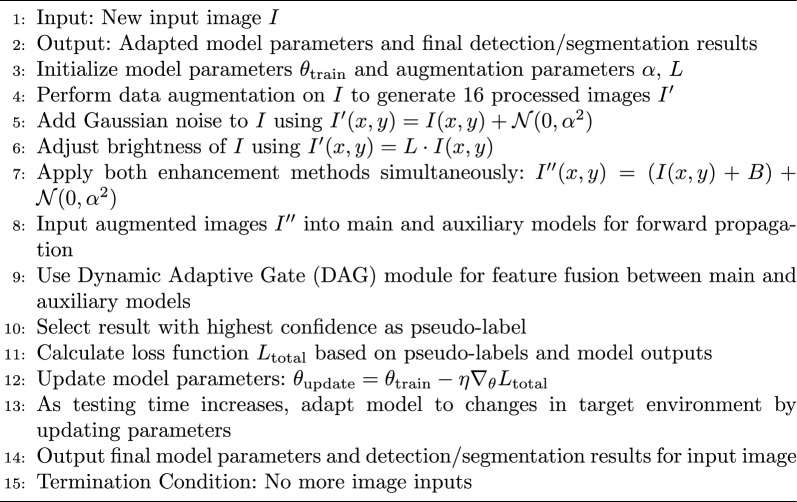
Test-Time Adaptation

Within the multi-task learning framework, by minimizing the total loss $\mathcal {L}_{total}$, the main model and the auxiliary model collaborate to optimize and enhance their understanding and processing capabilities of input images. The total loss $\mathcal {L}_{total}$ combines the losses of the main model and the auxiliary model, reflecting their performance on their respective tasks. To achieve this goal, we employ the gradient descent algorithm to update the model’s parameters. The gradient update formulas are as follows:

28 $$\begin{aligned} & \theta _{main} = \theta _{main} - \eta \nabla _{\theta _{main}} \mathcal {L}_{total}, \end{aligned}$$

29 $$\begin{aligned} & \theta _{aux} = \theta _{aux} - \eta \nabla _{\theta _{aux}} \mathcal {L}_{total}, \end{aligned}$$

where $\theta _{main}$ and $\theta _{aux}$ represent the parameters of the main model and the auxiliary model, respectively, $\eta$ is the learning rate, controlling the step size of the parameter update. The gradients $\nabla _{\theta _{main}} \mathcal {L}_{total}$ and $\nabla _{\theta _{aux}} \mathcal {L}_{total}$ are the gradients of the total loss $\mathcal {L}_{total}$ with respect to the parameters of the main model and the auxiliary model, respectively. These gradients indicate how to adjust the parameters to minimize the total loss.

Through the multi-task learning framework, MT-DAM not only addresses the challenge of balancing accuracy and generalization faced by single-task models but also enhances the complementarity between object detection and image segmentation tasks. Traditional single-task models often focus excessively on a specific task, leading to limitations when handling complex visual tasks. MT-DAM overcomes this challenge by sharing low-level features and leveraging the strengths of different tasks. The object detection task and the image segmentation task provide the model with different visual information at global and local levels, respectively. Object detection offers precise target locations for the image segmentation task, while image segmentation enhances the accuracy of object detection through fine processing at the pixel level. Therefore, the collaborative optimization between these two tasks not only improves the object detection model’s ability to recognize objects in complex scenes but also enhances the precision of image segmentation in handling edges and details. This mutual reinforcement makes MT-DAM more robust in dealing with complex visual tasks and capable of adapting to various complex scenes and environmental changes. We believe that the MT-DAM model not only introduces new concepts to the field of multi-task learning but also offers technological breakthroughs for visual tasks in practical applications.

## Results analysis

### Experiment setting

Our experiment is based on PyTorch 2.2.2 + CUDA 12.1 and is trained using NVIDIA GeForce RTX 4090. The training parameters are set to epochs=50, input image size=416, initial learning rate $\epsilon _{\text {init}}$=0.05, $\epsilon _{\text {final}}$=0.1, batch size=32, the strong augmenter retains 3 batches of images, with a learning rate of 0.01, and the adaptive gating module has a learning rate of 0.02.

### Dataset

Our dataset consists of 12,205 images from the IP102 dataset, supplemented by 4,060 images we collected ourselves, totaling 16,265 images across 53 classes. These are divided into a training set of 11,840 images, a validation set of 2,212 images, and a test set of 2,213 images. Neither the training set nor the validation set has been processed; the test set involves randomly fusing the original images with different weather images to form target environment images for testing the model’s cross-domain performance.

### Ablation experiment

**Table 1:** Ablation Experiment Results in the Original Environment

Strong augmenter	Weak augmenter	MT-DAM	Precision $\uparrow$	Recall $\uparrow$	mAP50 $\uparrow$	mAP50-95 $\uparrow$
✗	✗	✗	0.681	0.671	0.714	0.481
$\checkmark$	✗	✗	0.694	0.708	0.739	0.497
✗	$\checkmark$	✗	0.683	0.692	0.727	0.492
✗	✗	$\checkmark$	0.684	0.720	0.746	0.504
$\checkmark$	$\checkmark$	✗	0.714	0.753	0.768	0.511
$\checkmark$	✗	$\checkmark$	0.708	0.779	0.782	0.532
✗	$\checkmark$	$\checkmark$	0.716	0.758	0.774	0.518
$\checkmark$	$\checkmark$	$\checkmark$	0.725	0.771	0.790	0.539

**Table 2:** Ablation Experiment Results in the Cross-Domain Environment

Strong augmenter	Weak augmenter	MT-DAM	Precision $\uparrow$	Recall $\uparrow$	mAP50 $\uparrow$	mAP50-95 $\uparrow$
✗	✗	✗	0.431	0.402	0.376	0.239
$\checkmark$	✗	✗	0.478	0.463	0.477	0.281
✗	$\checkmark$	✗	0.474	0.439	0.438	0.263
✗	✗	$\checkmark$	0.506	0.484	0.503	0.296
$\checkmark$	$\checkmark$	✗	0.502	0.478	0.494	0.289
$\checkmark$	✗	$\checkmark$	0.526	0.503	0.524	0.311
✗	$\checkmark$	$\checkmark$	0.514	0.507	0.519	0.305
$\checkmark$	$\checkmark$	$\checkmark$	0.538	0.518	0.537	0.317

The results showing in Tables 1 and 2 that the influence of eight distinct component combinations on model performance markedly differs between the original and cross-domain environments. Specifically, in the original environment, the introduction of the strong augmenter alone leads to a significant improvement in metrics, particularly in precision and recall, which are improved to 0.694 and 0.708, respectively. In comparison, the weak augmenter modestly improves recall but with a more limited augment. The MT-DAM, excels in enhancing recall and mAP metrics, achieving 0.720 and 0.746. Furthermore, when the strong and weak augmenters are combined, all metrics are further elevated; most notably, when MT-DAM is concurrently employed with augmenters, the performance gains are most pronounced, with precision, recall, mAP50, and mAP50–95 peaking at 0.725, 0.771, 0.790, and 0.539. In the cross-domain environment, the baseline initial performance is relatively low. However, the augmenters and MT-DAM can significantly improve precision, recall, mAP50, and mAP50-95, achieving 0.538, 0.518, 0.537, and 0.317. Notably, the strong augmenter demonstrates a particularly significant improvement across all metrics in the cross-domain environment, while the MT-DAM stands out in enhancing recall and mAP metrics. Although the weak augmenter’s effect is relatively minor, it still exhibits potential for improvement in specific metrics. In conclusion, the synergistic application of these three components can maximize the model’s performance across varying environments.

### Comparative experiment

When evaluating the performance of object detection models, we typically employ the precision of the detection boxes (Precision), recall rate (Recall), and mean Average Precision (mAP50 and mAP50-95) at IoU thresholds of 50% and 50-95% as key metrics. These indicators comprehensively reflect the accuracy and completeness of the model in locating target objects. For image segmentation models, we also use these indicators, but they pertain to the segmented mask, that is, the segmentation results output by the model. These indicators can assess the segmentation precision and completeness at the pixel level, thereby providing a comprehensive evaluation framework for image segmentation tasks.

**Table 3:** Accuracy of the baseline model’s object detection and image segmentation tasks

Original environment	Precision$\uparrow$	Recall$\uparrow$	mAP50$\uparrow$	mAP50-95$\uparrow$
YOLOv9c	0.681	0.671	0.714	0.481
YOLOv9e	0.658	0.701	0.733	0.496
YOLOv9c-seg	0.613	0.606	0.607	0.507
YOLOv9e-seg	0.623	0.613	0.611	0.511

Target environment	Precision$\uparrow$	Recall$\uparrow$	mAP50$\uparrow$	mAP50-95$\uparrow$
YOLOv9c	0.431	0.402	0.376	0.239
YOLOv9e	0.457	0.53	0.481	0.313
YOLOv9c-seg	0.478	0.366	0.362	0.291
YOLOv9e-seg	0.461	0.415	0.399	0.321

**Table 4:** Accuracy of the baseline model’s object detection and image segmentation tasks with DynamicDA

DynamicDA in original environment	Precision$\uparrow$	Recall$\uparrow$	mAP50$\uparrow$	mAP50-95$\uparrow$
YOLOv9c	0.714	0.753	0.768	0.511
YOLOv9e	0.7	0.73	0.764	0.52
YOLOv9c-seg	0.658	0.683	0.651	0.527
YOLOv9e-seg	0.668	0.635	0.644	0.533

DynamicDA in target environment	Precision$\uparrow$	Recall$\uparrow$	mAP50$\uparrow$	mAP50-95$\uparrow$
YOLOv9c	0.502	0.478	0.494	0.289
YOLOv9e	0.529	0.562	0.541	0.334
YOLOv9c-seg	0.528	0.421	0.454	0.328
YOLOv9e-seg	0.533	0.438	0.459	0.344

By examining the data in Table 3 and 4, we find that after adopting dynamic data augmentation technology, the performance of the baseline model in both the target environment and the original environment has been significantly improved. Specifically, in the original environment, the v9c model achieved increases of 3.3%, 8.2%, 5.4%, and 3% in precision, recall, mean average precision (mAP50), and mean average precision (mAP50-95), respectively. Similarly, the v9e model also improved in these key indicators by 4.2%, 2.9%, 3.1%, and 2.4%, respectively. When we turn our attention to the target environment, these improvements become even more pronounced: the corresponding indicators of the v9c model increased by 7.1%, 7.6%, 11.8%, and 5%, while the v9e model also made significant progress with increases of 7.2%, 3.2%, 6%, and 2.1%. These significant figures not only confirm the effectiveness of dynamic data augmentation technology in improving the detection accuracy of models but also reveal its potential in enhancing the model’s cross-domain capabilities. The performance of the model in the target environment has been significantly enhanced, which is particularly striking, just by means of image enhancement.

In addition, we should not overlook the subtle but important improvements brought about by dynamic augmentation technology in the original environment. These improvements indicate that even in an environment where the model has already adapted, we can still enhance the performance of the model by further optimizing dynamic augmentation technology, making its detection results more accurate and stable. This enhancement is attributed to the ability of dynamic augmentation technology to increase data diversity during model training, thereby enhancing the model’s generalization ability to various types of data.

**Table 5:** Accuracy of the MT-DAM’s object detection and image segmentation tasks without DynamicDA

Original environment	Precision$\uparrow$	Recall$\uparrow$	mAP50$\uparrow$	mAP50-95$\uparrow$
MT-DAM(v9c)	0.684	0.72	0.746	0.504
MT-DAM(v9e)	0.701	0.713	0.745	0.507
MT-DAM(v9c-seg)	0.624	0.651	0.638	0.517
MT-DAM(v9e-seg)	0.669	0.631	0.639	0.523

Target environment	Precision$\uparrow$	Recall$\uparrow$	mAP50$\uparrow$	mAP50-95$\uparrow$
MT-DAM(v9c)	0.506	0.484	0.503	0.296
MT-DAM(v9e)	0.514	0.551	0.549	0.343
MT-DAM(v9c-seg)	0.507	0.438	0.481	0.346
MT-DAM(v9e-seg)	0.509	0.447	0.497	0.372

By conducting an in-depth analysis of the data in Table 3 and 5, we observe an interesting phenomenon: although the introduction of the dynamic adaptive gating (DAG) mechanism in the multi-task dynamic adaptation model (MT-DAM) did not significantly enhance the model’s performance in the original environment, with the v9c model showing only marginal improvements of 0.3%, 4.9%, 3.2%, and 2.3% in various metrics, a shift in perspective to the target environment reveals that this strategy brought about significant performance improvements. For the v9c model, the performance enhancements in the target environment were notably 7.5%, 8.2%, 12.7%, and 5.7%. Similarly, the v9e model also showed excellent performance in the target environment, with improvement margins of 5.7%, 2.1%, 6.8%, and 3%.

This phenomenon indicates that while the information exchange between the main model and the auxiliary model in the familiar original environment may not have led to the expected significant performance improvements, in the target environment, such information exchange effectively promoted the knowledge complementarity between the two models. Through the DAG mechanism, the main model and the auxiliary model can learn from each other and share beneficial feature representations, thereby achieving more accurate detection and classification in the target environment. This complementarity not only enhances the model’s adaptability to the target environment but also demonstrates the potential of dynamic adaptive gating technology in promoting the model’s cross-domain generalization capabilities.

**Table 6:** Accuracy of the CDPD’s object detection and image segmentation tasks

Original environment	Precision$\uparrow$	Recall$\uparrow$	mAP50$\uparrow$	mAP50-95$\uparrow$
CDPD(v9c)	0.725	0.771	0.79	0.539
CDPD(v9e)	0.737	0.728	0.772	0.523
CDPD(v9c-seg)	0.669	0.685	0.662	0.535
CDPD(v9e-seg)	0.667	0.647	0.655	0.531

Target environment	Precision$\uparrow$	Recall$\uparrow$	mAP50$\uparrow$	mAP50-95$\uparrow$
CDPD(v9c)	0.538	0.518	0.537	0.317
CDPD(v9e)	0.553	0.559	0.577	0.361
CDPD(v9c-seg)	0.544	0.493	0.508	0.362
CDPD(v9e-seg)	0.548	0.474	0.515	0.381

**Fig. 5:**
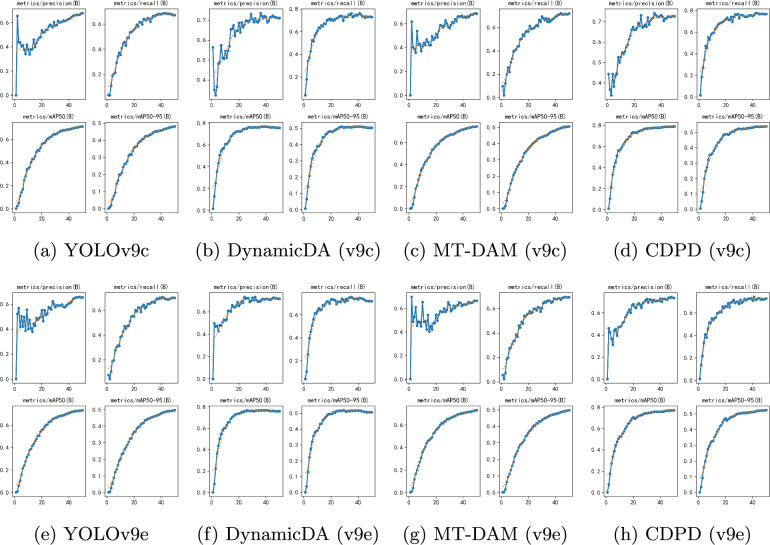
Comparison of precision graphs for various methods trained in the original environment. The figure illustrates the significant improvement in model convergence rates after dynamic augmentation and the changes in model precision under different methods

**Fig. 6:**
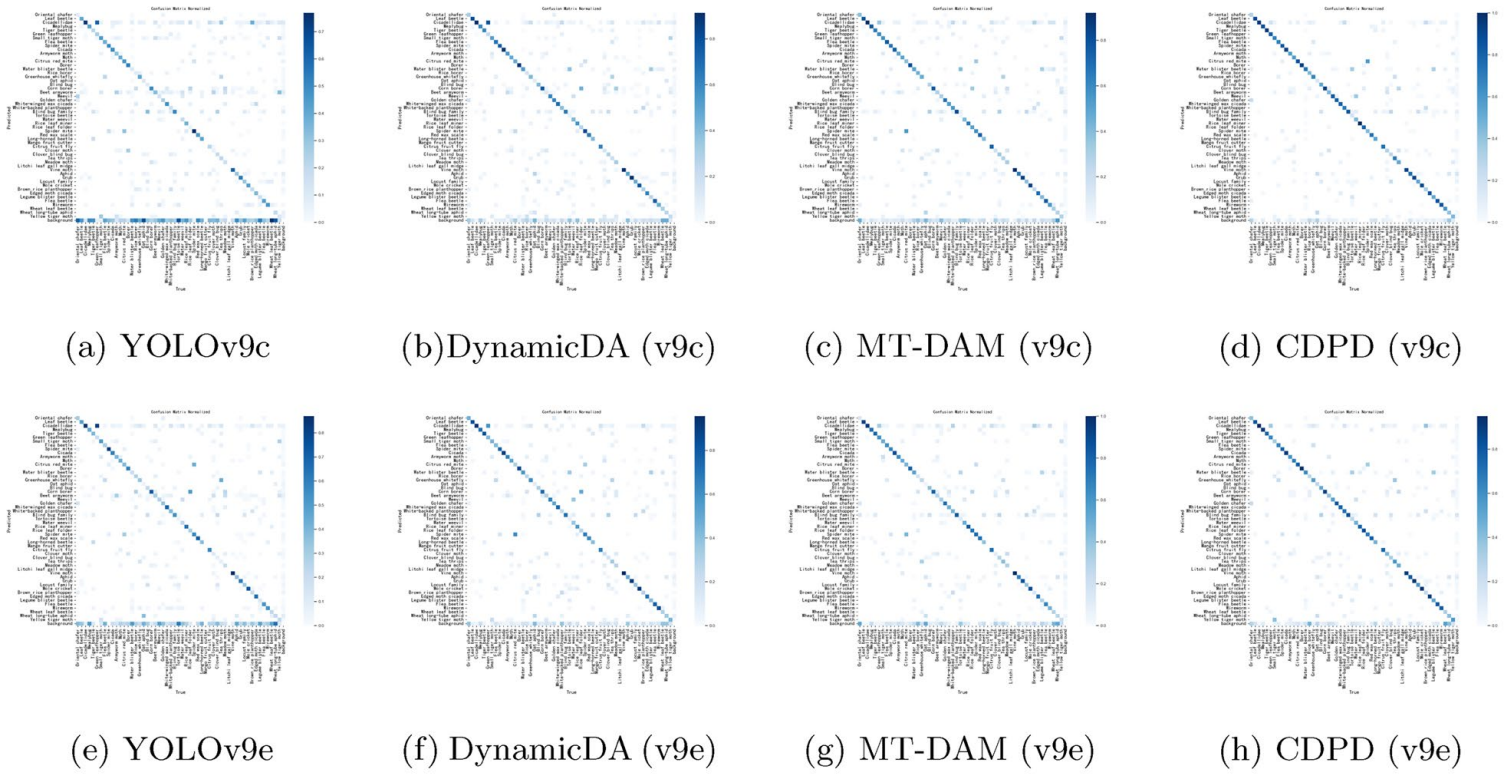
The confusion matrix demonstrates that the CDPD method exhibits a lower error rate in the target environment, performs superiorly compared to single-module applications, and enhances the model’s cross-domain adaptability and generalization ability by combining features from two modules

Upon meticulous analysis of the data in Table 3 and 6, we have arrived at a series of encouraging conclusions. For the method based on the v9c model, we observed a significant enhancement in performance within the original environment, characterized by an improvement of 4.4% in precision, 10% in recall, 7.6% in mean average precision (mAP50), and 5.8% in mean average precision (mAP50-95). More thrillingly, in the target environment, these enhancements were even more pronounced, reaching 10.7%, 11.6%, 16.1%, and 7.8% respectively. For the method based on the v9e model, although the performance improvements in the original environment were more moderate, they still achieved increases of 7.9%, 2.7%, 3.9%, and 2.7% respectively; and in the target environment, the performance improvements were more significant, reaching 9.6%, 2.9%, 9.6%, and 4.8% respectively. These data not only confirm that our method can significantly enhance the model’s detection capabilities in the target environment but also, to a certain extent, improve the model’s performance in the original environment.

Our analysis further reveals the key role of dynamic data augmentation technology. This technology effectively simulates the complexity of the target environment by introducing augmented images with different environmental characteristics during the model training period, thereby enabling the model to adapt more quickly to new environments and enhance its generalization capabilities. Additionally, the joint training strategy based on the dynamic adaptive gating module (DAG) achieves effective knowledge transfer between the main task and the auxiliary task by intelligently controlling the flow of information and feature fusion. In this study, the main task is positioned in object detection, while the auxiliary task focuses on image segmentation. This cross-task joint training not only improves the model’s precision in object detection but also enhances the robustness of object detection by leveraging the detailed features of image segmentation.

From Fig. 5 (a), (b) and (e), (f), we observe that after dynamic augmentation, the model’s convergence rate significantly improved. This acceleration means the model can reach a stable state more quickly during the training process, thereby reducing training time and increasing efficiency. Further analysis of the confusion matrices in Fig. 6 (a), (b) and (e), (f) reveals that, aside from a few indistinguishable classes, the error rates for other classes have significantly decreased. This indicates that the model’s recognition ability for most classes has improved, reducing misclassifications.

**Table 7:** CDPD method’s precision variation during testing

t	Precision$\uparrow$	Recall$\uparrow$	mAP50$\uparrow$	mAP50-95$\uparrow$
CDPD(v9c)	0.538	0.518	0.567	0.317
CDPD(v9e)	0.553	0.559	0.577	0.361
CDPD(v9c-seg)	0.544	0.493	0.508	0.362
CDPD(v9e-seg)	0.548	0.474	0.515	0.381

15	Precision$\uparrow$	Recall$\uparrow$	mAP50$\uparrow$	mAP50-95$\uparrow$
CDPD(v9c)	0.649	0.627	0.645	0.412
CDPD(v9e)	0.648	0.632	0.661	0.437
CDPD(v9c-seg)	0.657	0.605	0.582	0.451
CDPD(v9e-seg)	0.639	0.613	0.597	0.468

30	Precision$\uparrow$	Recall$\uparrow$	mAP50$\uparrow$	mAP50-95$\uparrow$
CDPD(v9c)	0.696	0.694	0.705	0.479
CDPD(v9e)	0.705	0.696	0.724	0.493
CDPD(v9c-seg)	0.699	0.663	0.627	0.506
CDPD(v9e-seg)	0.697	0.672	0.635	0.517

45	Precision$\uparrow$	Recall$\uparrow$	mAP50$\uparrow$	mAP50-95$\uparrow$
CDPD(v9c)	0.711	0.713	0.723	0.493
CDPD(v9e)	0.721	0.709	0.738	0.513
CDPD(v9c-seg)	0.707	0.681	0.645	0.505
CDPD(v9e-seg)	0.709	0.717	0.653	0.521

From Fig. 5 and 6 (a), (c) and (e), (g), it can be observed that although the convergence speed did not significantly improve after adding the auxiliary model, the confusion matrices show a significant decrease in error rates, further demonstrating that our MT-DAM strategy effectively enhances the model’s robustness in the target environment. By analyzing the confusion matrices in Fig. 5 and 6 (a), (d) and (e), (h), it is evident that the CDPD method shows superior performance compared to the application of a single module alone. Observing the training accuracy curves reveals that the CDPD method combines the advantages of both modules, improves the model’s convergence speed, and ultimately reaches a stable state without performance degradation due to overfitting. These results fully prove that the CDPD method effectively integrates the characteristics of both modules, endowing the model with excellent cross-domain adaptability and generalization capabilities.

From Table 7, it can be observed that as the training time increases, the model’s predictive accuracy also improves, gradually approaching the baseline values in the original environment. This phenomenon indicates that the testing-time adaptation strategy we proposed is effective and significantly enhances the model’s adaptability in the target environment. Specifically, as the training period is extended, the model’s ability to capture the complexity and dynamic changes of the target environment is strengthened, thereby improving the model’s generalization performance. Furthermore, the stability of the model in the target environment is also validated, which further confirms the effectiveness of the testing-time adaptation method.

**Table 8:** The performance of various methods in an environment that changes over time (mAP50)

$${\textbf {Time}} \hspace{6em} \overrightarrow{\phantom{LLLLLLLLLLLLLLLLLLLLLLLLLLLLLLLLLLLLLLLLLLLLLLLLLLLLLLLLLLLLLLLLLLLLLLLLLLLLLLLLLLLLL}}$$								
	BR	DA	GN	FO	RA	SN	SP	Avg
YOLOv9c	0.527	**0.658**	0.635	0.494	0.552	0.459	0.597	0.560
BN	0.516	0.563	0.595	0.453	0.436	0.397	0.454	0.488
PL	0.397	0.382	0.425	0.311	0.305	0.277	0.275	0.339
TENT	0.414	0.397	0.413	0.289	0.267	0.254	0.233	0.324
CoTTA	0.527	0.623	0.629	**0.567**	0.569	0.497	0.556	0.567
CDPD(Our)	**0.534**	0.627	**0.646**	0.562	**0.628**	**0.567**	**0.663**	**0.604**

The bolded data in the table represents the highest value in the column

**Fig. 7:**
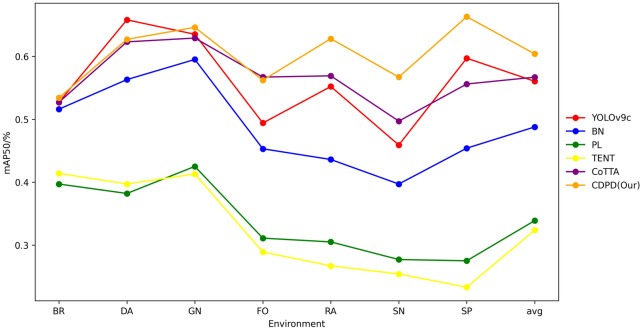
The performance of various methods in an environment that changes over time (mAP50)

**Fig. 8:**
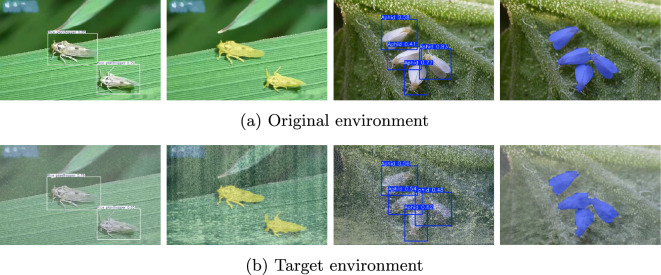
The performance of the primary model and the auxiliary model in the original and target environments. The target environment encompasses fog, rain, snow, and frost.The primary model focusing on object detection tasks and the auxiliary model dedicated to image segmentation. They complement each other and enhance overall performance

Based on Table 8 and Fig. 7, the CDPD method stands out with the value of 0.604 in the average mAP50 metric, confirming its superior performance in cross-domain pest detection tasks. Observations from the charts reveal that the accuracy of the PL and TENT methods continuously declines in later stages, indicating an accumulation of errors during processing, leading to the forgetting of correct knowledge. Similarly, the BN method also shows a noticeable decrease in performance when transitioning from the GN (Gaussian noise) scenario to the SN (snow) scenario. In contrast, although the accuracy of various methods fluctuates in changing environments, the CDPD method reached the values of 0.628, 0.567, and 0.663 in the RA (rain), SN (snow), and SP (salt and pepper noise) environments. This result indicates that the CDPD method is capable of handling most cross-domain detection tasks, which is crucial for detection in actual dynamically changing environments. Figure 8 further illustrates the performance of the primary model (focused on object detection) and the auxiliary model (focused on image segmentation) in the original and target environments, including rain, snow, and others, validating the robustness of model collaboration in dynamic environments and supporting the superior performance of the CDPD method.

**Fig. 9:**
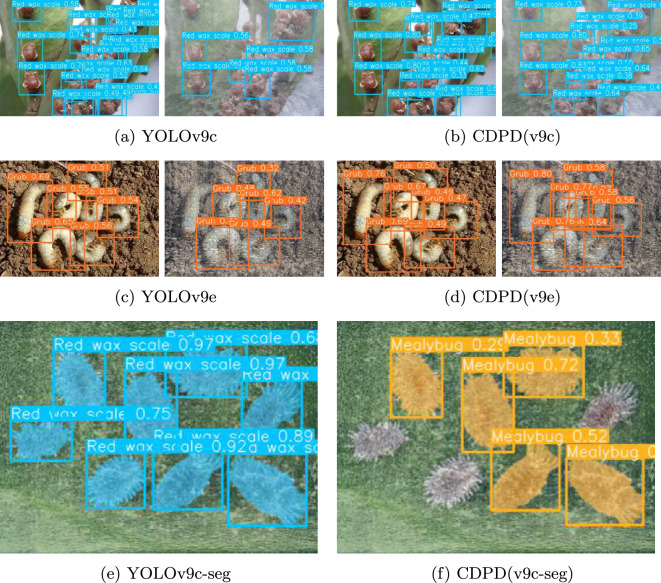
Multi-task Object Detection and Image Segmentation Effect Images. The CDPD method has effectively enhanced the confidence in object detection and image segmentation tasks compared to the baseline, within the original environment. Moreover, it has addressed the issues of false positives and missed detections in the target environment, achieving a complete accuracy in detection outcomes

From the results observed in Fig. 9, our model demonstrated exceptional performance in both the original and target environments, particularly in multi-object detection tasks. It not only maintained stable detection effects in the original environment but also achieved higher detection accuracy in complex scenes compared to the original model, indicating the model’s good generalization ability. This ability allows the model to maintain stable detection effects when facing different environmental conditions.

In summary, the model proposed in this study has achieved significant technological breakthroughs and has shown excellent practicality in real-world applications. Without acquiring knowledge of the target environment, the model enhanced by introducing DynamicDA alone increased the mean Average Precision (mAP50) in the target environment by 11.8%. Furthermore, relying solely on the joint training of the multi-task model (MT-DAM) without using augmented images, the mAP50 of the model in the target environment improved by 12.7% compared to the original model. The combined use of DynamicDA and MT-DAM in the target environment achieved even more significant improvements, reaching 16.1%. In addition, in the original environment, mAP50 also achieved a 7.6% increase, reaching 0.79. Through testing-time adaptation methods, the model’s mAP50 in the target environment reached 0.738, close to the level of 0.79 in the original environment. These results fully prove that our method can effectively improve the model’s accuracy in the target environment and strengthen its ability to adapt to different environments, even in the absence of knowledge about the target environment. a.

## Discussion

In this study, we introduce the CrossDomain-PestDetect (CDPD) method, which leverages YOLOv9 and a Test-Time Adaptation (TTA) framework to address the challenges of cross-domain pest detection in complex agricultural environments. The CDPD method comprises three key components: Dynamic Data Augmentation (DynamicDA), Dynamic Adaptive Gate (DAG) module, and MultiTask Dynamic Adaptation Model (MT-DAM). DynamicDA enhances the model’s adaptability by generating diverse and category-consistent augmented images through a learnable strong augmenter and a weak augmenter. The DAG module optimizes feature fusion between the main object detection model and the auxiliary image segmentation model by dynamically controlling information flow based on learned parameters, ensuring that only useful and accurate information is shared. The MT-DAM facilitates effective knowledge transfer between detection and segmentation tasks, thereby improving the model’s robustness and accuracy. Experimental results demonstrated that CDPD significantly outperforms traditional baseline models, achieving higher Precision, Recall, mean Average Precision (mAP50), and mean Average Precision (mAP50–95) in both original and target environments. These findings validate the effectiveness of our approach in enhancing cross-domain detection capabilities, particularly within target environments, and highlight the potential of dynamic adaptive gating in feature fusion.

However, the study is not without limitations. The computational overhead associated with the joint training of the main and auxiliary models, along with the test-time adaptation, may pose a challenge for real-time applications. This is an important consideration for the practical deployment of the CDPD method in resource-constrained environments.In future research endeavors, we aim to delve into the exploration of lightweight model designs to mitigate computational expenses, while ensuring the efficiency and accuracy of the CDPD method within resource-constrained settings.

## Conclusions

The CDPD method stands as a robust solution for pest detection in dynamic agricultural settings. It not only improves detection accuracy but also enhances the model’s ability to generalize across different environments. The significance of this study lies in its potential to revolutionize pest management practices by enabling more timely and effective interventions.

Despite the promising results, this study has certain limitations. The joint training of main and auxiliary models, along with the test-time adaptation process, leads to increased training and inference times. Future research should focus on developing lighter model architectures and further optimizing feature fusion strategies to enhance computational efficiency without compromising performance. Additionally, expanding the diversity of data augmentation techniques and refining loss functions could further improve the model’s generalization and adaptability to diverse and dynamic environments. By addressing these areas, the CDPD method can be further optimized, promoting its application in practical pest detection scenarios and contributing to advancements in multi-task learning and cross-domain adaptation in deep learning models. Continuous technological innovation and optimization are essential to fully realize the potential of deep learning models in complex and variable real-world environments.

## Data Availability

This study analyzed a combination of publicly available datasets and data collected by the authors. The publicly available datasets can be accessed at [https://github.com/xpwu95/IP102]. If you want to request the complete dataset, please email the corresponding author.
